# Qualitative microbiome profiling along a wastewater system in Kampala, Uganda

**DOI:** 10.1038/s41598-019-53569-5

**Published:** 2019-11-22

**Authors:** Pierre H. H. Schneeberger, Samuel Fuhrimann, Sören L. Becker, Joël F. Pothier, Brion Duffy, Christian Beuret, Jürg E. Frey, Jürg Utzinger

**Affiliations:** 1 Department of Method Development and Analytics, Agroscope, Wädenswil, Switzerland; 2 Department of Virology, Spiez Laboratory, Federal Office for Civil Protection, Spiez, Switzerland; 3 Swiss Tropical and Public Health Institute, Basel, Switzerland; 4 University of Basel, Basel, Switzerland; 5 University Health Network, Toronto, Canada; 6 University of Toronto, Toronto, Canada; 7 Institute for Risk Assessment Sciences, Utrecht University, Utrecht, The Netherlands; 8 Institute of Medical Microbiology and Hygiene, Saarland University, Homburg/Saar, Germany; 9 Institute of Natural Resource Sciences, Zurich University of Applied Sciences, Wädenswil, Switzerland

**Keywords:** Water microbiology, Freshwater ecology, Wetlands ecology

## Abstract

Kampala, the capital city of Uganda, is rapidly expanding without adequate wastewater treatment facilities to accommodate the current estimated population of 1.68 million people. Hence, freshwater bodies and natural ecosystems around the city are heavily polluted with organic and inorganic contaminants. Yet, there is a paucity of data on pathogenic microorganisms, which potentially threatens health of local communities. We performed a qualitative microbial analysis using a whole metagenome sequencing approach encompassing over 150 gigabases of sequencing data to characterize the Nakivubo wastewater system, which includes a wastewater channel and surrounding wetlands. We found that microbial diversity is heterogeneous throughout the system and that three community state types could be differentiated. We showed the presence of various waterborne agents of gastrointestinal infections in humans, which were associated with leakage occurring around two locations along the wastewater channel. Our data indicate that the microbial decontamination capacity of the local wastewater treatment facility was insufficient at the time of sampling, and that several areas of the wetlands were contaminated with human pathogens, indicating that parts of the wetlands are potentially unsafe for urban agriculture.

## Introduction

Wastewater is a generic term encompassing any water which has been negatively modified by human activities^1^. Wastewater is a double-edged sword: on one hand, it represents a source of water which could potentially be (re)-utilized in various industries^2^; on the other hand, it is the source of multiple hazards for human health^3,4^. The biological risk associated with wastewater is particularly challenging to assess and control due to the diversity of waterborne agents, which might give rise to human disease, through a multitude of exposure pathways^5^. Current assessment of water contamination includes mainly conventional microbiological methods (e.g. culture of water samples on agar plates and counting of specific types of bacteria, such as total faecal coliforms or *Escherichia coli*), and to some extent, the use of molecular assays to identify specific microorganisms^6^. Wastewater is of particular relevance in low- and middle-income countries (LMICs), emphasized by rapid urbanization, profound demographic changes and tropical climate, which result in growing amounts of wastewater and, consequently, overloaded wastewater treatment infrastructures. These characteristics set the stage for the establishment of ecological niches with ideal growth conditions of potentially pathogenic microorganisms.

In the current study, we focus on a major wastewater system in Kampala, the capital of Uganda^7,8^. Kampala is located on the northern shores of Lake Victoria at an altitude of 1,140 m above sea level. The climate is tropical with precipitations throughout the year, mainly concentrated during two rainy seasons; the first occurring between March and May and the second one from October to November. With an estimated population of 1.68 million inhabitants in 2019 and an average annual population growth rate of 5.6%, Kampala is among the fastest growing cities worldwide^9,10^. Despite profound demographic changes and economic development, arising social and health-related challenges have yet to be fully addressed, as can be seen in the relatively moderate increase of funding in the field of water supply^11^. It is widely acknowledged that considerable population growth, coupled with rapid urbanization, put strains on existing wastewater infrastructures. Indeed, only approximately 10% of Kampala’s population is connected to a sewer, while most people are relying on on-site sanitation systems such as pit latrines and septic tanks^7,8,12^. Moreover, industrial development and urban farming have led to a reduction of wetland systems around the city that have previously served as natural wastewater treatment resources^8,12,13^. With a surface of 5.29 km^2^ and a total catchment area of over 40 km^2^ (ref.^14^), the Nakivubo wetland is the largest of a series of 12 wetland areas surrounding the city of Kampala. The Nakivubo wetland is divided by an old railway line with the area located north of the railway being composed mainly of drained wetland and the area located south of it composed mainly of floating wetlands. The Nakivubo wetland also serves for vegetable farming, with yams and sugarcane being the main cultivated crops. As farmers directly re-use wastewater for irrigation purposes, any health threats present in the water might negatively impact the farmers’ health and the safety of agricultural products grown in this area^15,16^. The Nakivubo wetland area is also subjected to flooding events, especially during the rainy season, and this puts an estimated 12,000 individuals in the surrounding slums at risk of direct contact with wastewater^13,17^. Thus far, several studies highlighted the potential risk of exposure to wastewater on human health^15,16,18–21^. A previous study by Fuhrimann *et al*. (2015) reported that the counts of colony-forming units (CFUs) of both *E. coli* and *Salmonella* spp. along the main wastewater treatment system in Kampala, including the Nakivubo channel and wetlands, were above the thresholds set by the World Health Organization (WHO) for unrestricted use for irrigation in agriculture^22^.

Several studies investigating the wastewater microbiome have been published^23,24^, but they generally involve partial 16S rRNA gene sequencing, a technique which usually does not enable microbial characterization at the lowest taxonomic levels (e.g. species/strains). Hence, detailed phylogenetic and microbiological information on the exact composition of pathogenic organisms is scarce, especially in rapidly growing urban areas of sub-Saharan Africa^21,25^ such as Kampala.

Here, we employed a shotgun metagenomics approach on water samples collected throughout the Nakivubo system^8^ to qualitatively characterize the microbiological composition of this dynamic environment and to determine potential health consequences for the exposed population. The novelty of the study lies in the use of deep metagenomics in a resource-constraint setting to provide an in-depth representation of the effective microbial contamination, and potential human health specific risks, along a populated and economically important water body. We provide a system-wide analysis of the Nakivubo system by: (i) grouping samples with regards to their microbial profiles; (ii) characterizing each group’s specificities; and (iii) further focusing our analysis on the distribution of potentially pathogenic microbes and on their relationships with wastewater contamination.

## Results

### Sequencing profiles

Measured DNA concentrations in a volume of 60 µl ranged from 1.7 ng/µl to 19.2 ng/µl for a total DNA minimum range of 106.2 ng to 1,152 ng per sample. A total of 1,240,255,828 reads were sequenced for the 22 samples with an average of 56,275,265 reads per sample. Using the GSMer database, an average of 11,588 highly specific matches were found for each sample. Detailed results are provided in Supplementary Table S1.

### Spatial relationships

**Figure 1:**
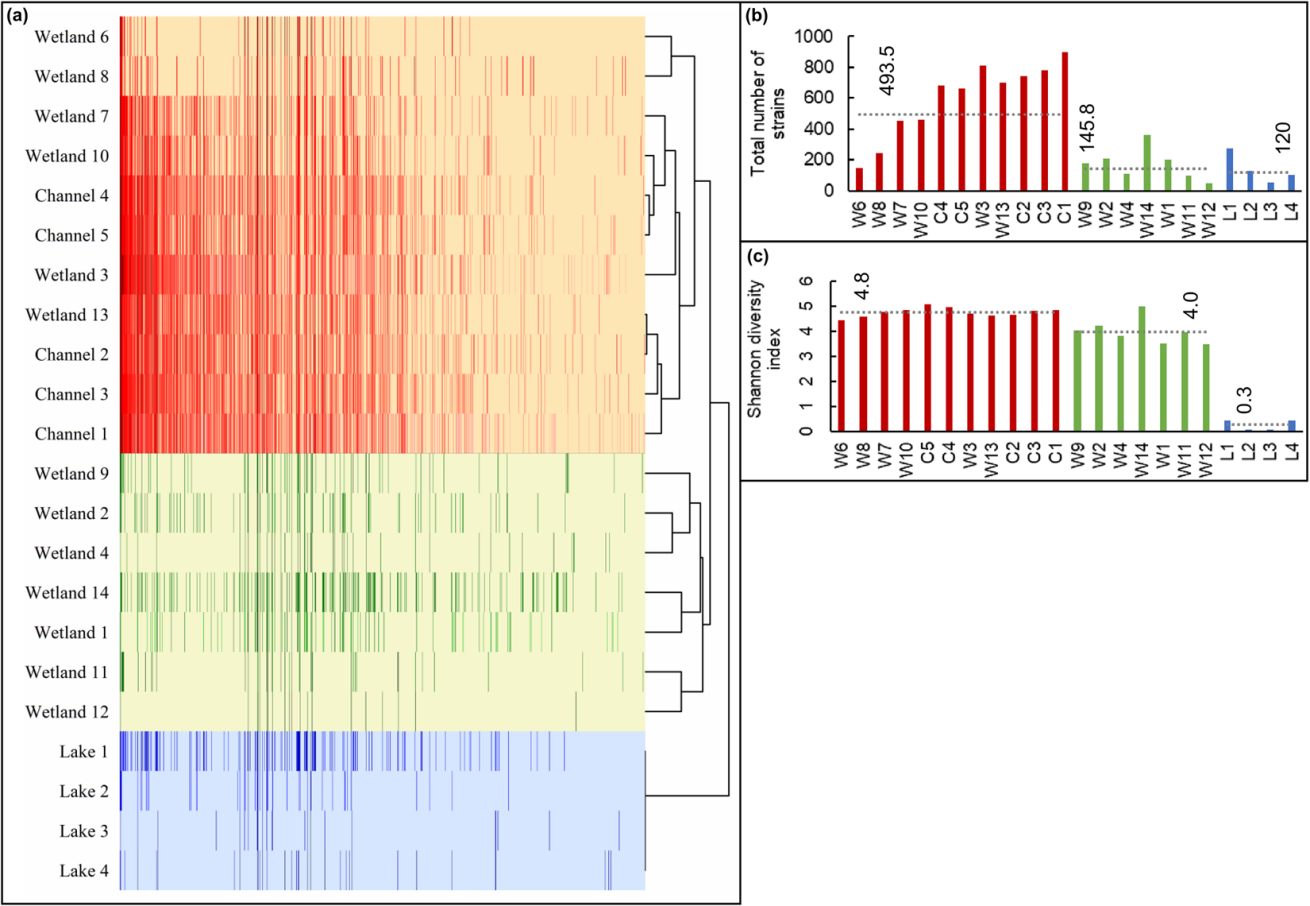
Sample-to-sample relationships. (**a**) Correlation-based hierarchical cluster analysis of water samples based on the relative abundance of bacterial strains, constructed using the group average method and a Spearman correlation matrix. The dendrogram shows the degree of similarity between the different samples (scale = 1). The three community state types (CST), which were selected for the rest of the study, are highlighted in orange (CST 1), green (CST 2) and blue (CST 3). (**b**) Total number of observed strains, per sample. The average cluster diversity is shown in dashed lines. C = channel; L = lake; W = wetland. (**c**) Shannon diversity index, per sample. The average value of the Shannon diversity per cluster is shown in dashed lines.

Using the taxonomic profiles derived from the comparison of the datasets with the GSMer database, we performed a hierarchical cluster analysis of all samples using a Spearman correlation matrix, as shown in Fig. 1a. All samples collected on Lake Victoria (L1–L4) clustered together in the most distant ramification that we refer to as community state type (CST) 3. The rest of the samples are separated in two distinct branches, namely: (i) the samples collected at the channel locations (C1–C5) together with samples collected at six wetland locations (W3, W6, W7, W8, W10 and W13) and subsequently referred to as CST 1; and (ii) the remaining six wetland samples (W1, W2, W4, W11, W12 and W14) that we subsequently refer to as CST 2. As shown in Fig. 1b, the average number of bacterial strains found in CST 1 (*n* = 493.5) was significantly higher than the average number of strains found in CST 2 (*t*-test, *P* = 4.3 × 10^−4^) and CST 3 (*t*-test, *P* = 0.003). In contrast, the difference between CST 2 and CST 3 was not statistically significant. Similarly, the mean Shannon diversity index (SDI), an indicator taking into account both abundance and evenness within a sample, was significantly lower in CST 2 (*t*-test, *P* = 0.003) and CST 3 (*t*-test, *P* = 2.2 × 10^−15^) compared to CST 1 (Fig. 1c). The SDI of CST 2 was significantly different from CST 3 (*t*-test, *P* = 2.4 × 10^−7^).

**Figure 2:**
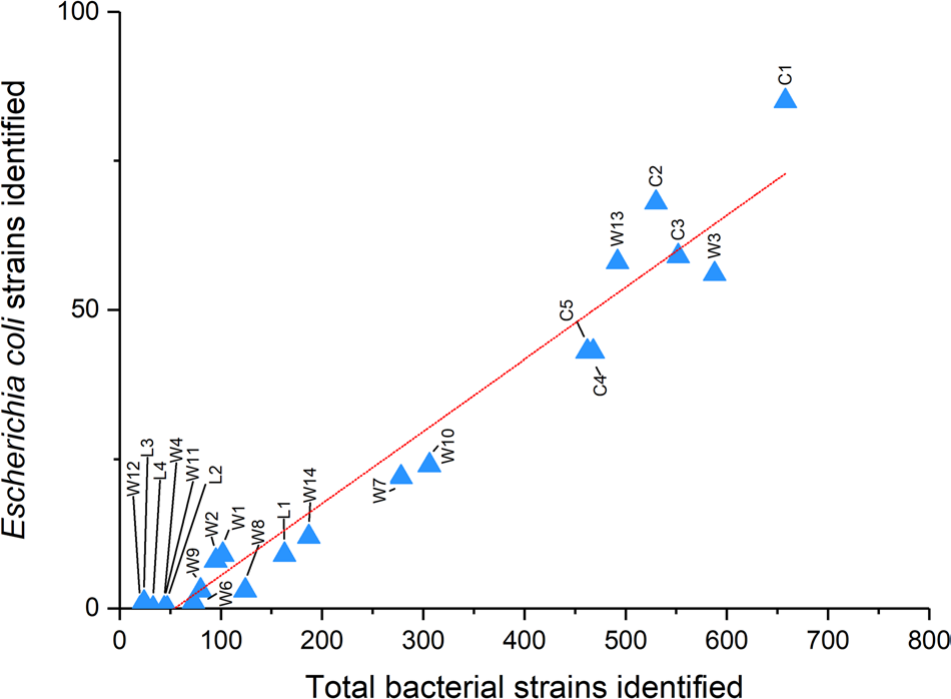
Linear regression analysis of *Escherichia coli* strains (ECS) and the total number of observed strains (NOS). This regression shows the relation between the intra-species diversity of *E*. *coli* and the total diversity among all bacteria (*R*^2^ = 0.958, *P* < 10^−4^). The total number of observed species can be estimated along the Nakivubo system using the following equation: NOS = 62.2 + 7.94 × ECS.

We further used the GSMer-derived profiles to compare the number of identified *E*. *coli* strains to the total amount of bacterial strains per sample, using a linear regression analysis (LRA), as shown in Fig. 2. We found a strong correlation between the diversity of *E*. *coli* species and that among all bacteria combined, indicating that the *E*. *coli* diversity may be used as a reliable indicator of overall bacterial diversity in this ecosystem.

In order to assess whether bacterial composition along the wastewater channel is linearly associated with the distance between each channel sample and the first channel sample (C1), we performed another LRA of distance from C1 against correlation with C1, as shown in Supplementary Fig. S1a. A strong relation between taxonomic correlation and the distance between channel samples was established, indicating that bacterial composition observed at C1 changes slowly, while moving away from this point along the Nakivubo channel. A final LRA was performed between channel and wetland samples from CST 1 in order to assess whether the bacterial composition of wetland samples is modified by a leakage at the closest channel sample (Supplementary Fig. S1b–g**)**. A strong correlation between taxonomic composition and the distance to the closest channel sample was found for samples W6, W8 and W10.

### Specificities of the community state types

**Figure 3:**
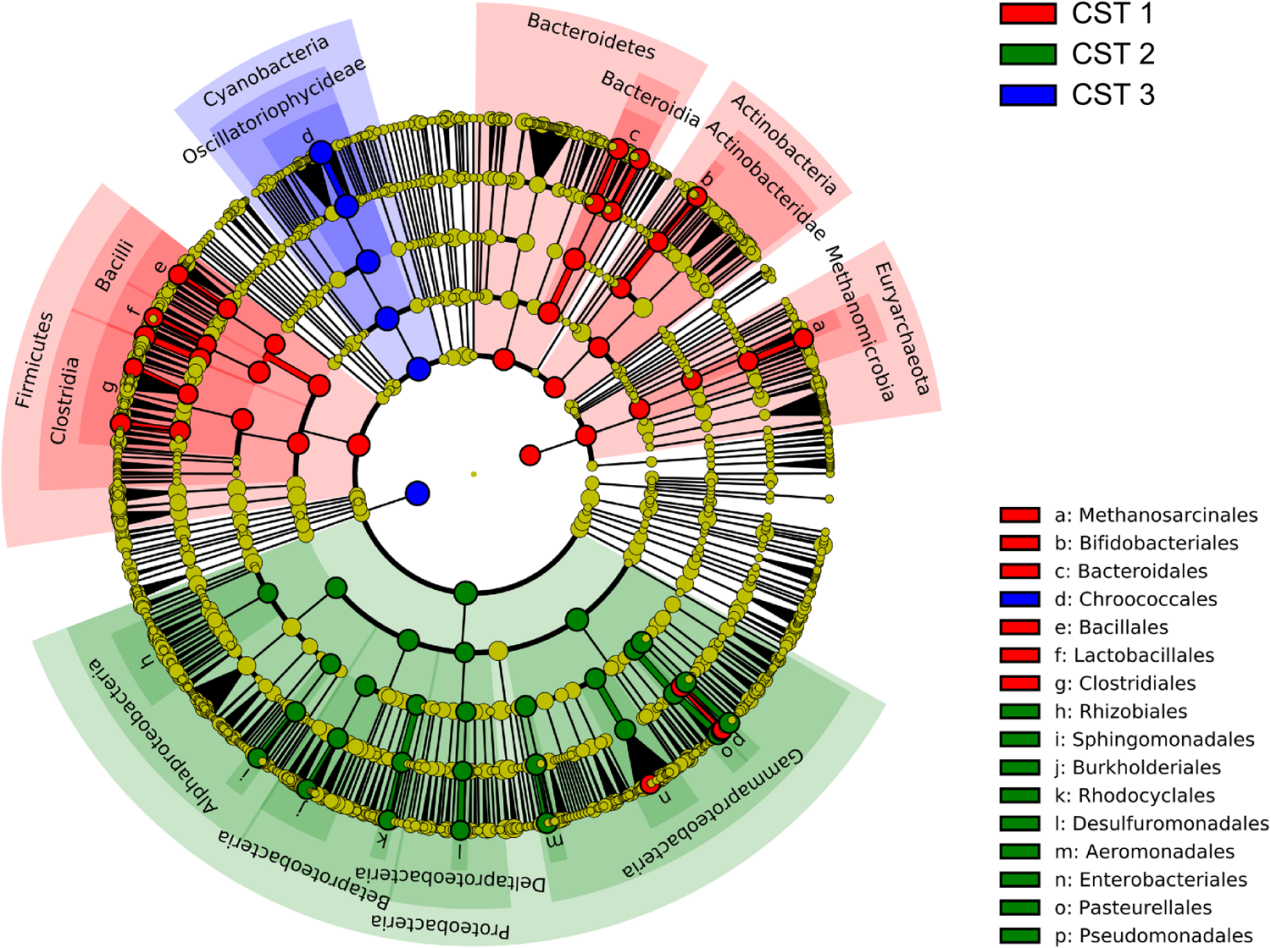
Cluster-related biomarkers. **(a)** The cladogram shows bacterial taxa which are significantly more abundant in each of the three community state types (*Q*-value < 0.01, linear discriminant analysis >4.0). For clarity, significant differences are shown only down to the taxonomical level of order. For a complete list of identified organisms, see Supplementary Table S3. Members from the Archaea and Bacteria domains are shown.

Bacterial profiles inferred from the GSMer database were compared, in order to highlight which bacterial taxa are more abundant in each of the three identified community state types (Fig. 3).

The bacterial composition of the CSTs showed various specificities (see Supplementary Table S2 for details). In brief, abundance differences included Gram-positive bacteria from the *Firmicutes* and *Actinobacteria* phyla, which were overabundant in samples presenting CST 1 in comparison to other types. The differences in *Clostridium* abundance included species from the *Blautia* and *Ruminococcus* genera, whereas differences in the *Bacilli* included species from the *Enterococcus*, *Streptococcus* and *Exiguobacterium* genera. *E*. *coli* and *Enhydrobacter aerosaccus* were also over-represented in CST 1. Overabundance of *Prevotella* and *Bacteroides* genera, within the Gram-negative *Bacteroidetes* phylum and of *Methanosaeta concilii* (Phylum *Euryarcaeota*) were additional characteristics of CST 1 samples. For CST 2, we highlighted the overabundance of one phylum, namely the *Proteobacteria*. This overrepresentation included members from the *Alpha*- (*Novosphingobium* spp.), *Beta*- (*Comamonas testosteroni* and *Dechloromonas aromatica*), *Delta*- (*Geobacter* spp.) and *Gammaproteobacteria* classes (*Pseudomonas*, *Acinetobacter*, *Pasteurella* and *Aeromonas* genera). CST 3 was characterized by the overabundance of *Cyanobacteria* with *Microcystis aeruginosa* being the dominant species.

**Figure 4:**
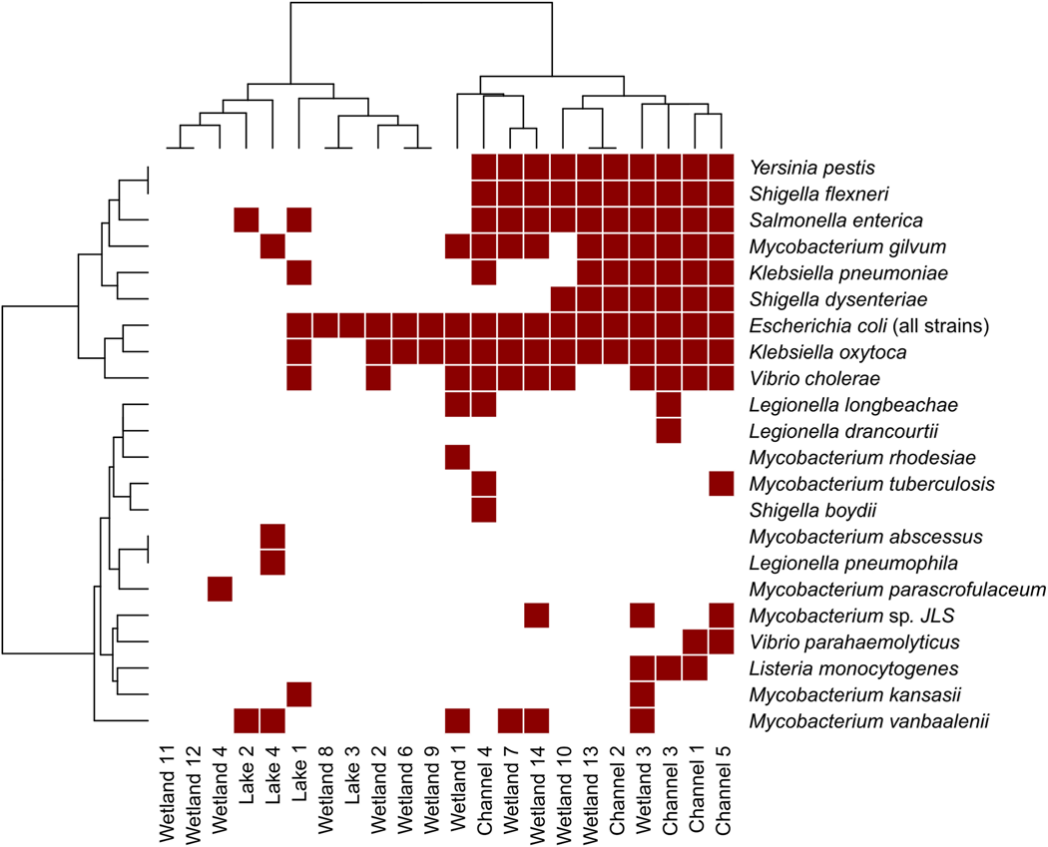
Heatmap of pathogen presence (red) or absence (white) across the Nakivubo system.

To estimate the potential risk of wastewater contamination on human health, we assessed the prevalence of a set of known waterborne bacterial pathogens throughout the Nakivubo system, which we refer to as the “pathobiome” (Fig. 4). The pathobiome-based clustering was similar to the clustering of the complete taxonomic profiles, except for samples W6 and W8, which were closer to samples presenting CST 2, while sample W14 was closer to samples presenting CST 1. The prevalence of these taxa is highest in samples collected directly in the wastewater channel (50%). The prevalence was significantly higher (Fisher’s exact test, *P* = 0.002) in wetland samples presenting CST 1 (29%) than in wetland and lake samples presenting CST 2 (14.2%) and CST 3 (14.7%). Some species, including *Klebsiella pneumoniae*, *Legionella dracourtii*, *Listeria monocytogenes*, *Mycobacterium tuberculosis*, *Shigella boydii*, *Shigella dysenteriae*, and *Vibrio parahaemolyticus* were found exclusively in samples presenting CST 1, indicating a close association between their presence and proximity to the wastewater channel. The overall richness of the pathobiome in samples presenting CST 1 was of 8.4 bacterial species, whereas samples presenting CST 2 and CST 3, had 3.14 and 3.25 species on average, respectively.

## Discussion

Using a metagenomics approach, we performed a qualitative analysis of the microbial composition of water samples taken along the major wastewater channel in Kampala, Uganda. The aim was to perform a system-wide microbial survey and to identify potential microbial health hazards. Our study is among the first investigations to apply deep shotgun sequencing with subsequent bacterial species/strain-level characterization in a sub-Saharan African setting.

There are several features of the Nakivubo system, which might influence the health of people living in the surrounding area. The first observation concerns bacterial diversity. Our data indicate that the system is composed of specific microbiological niches, represented by three CSTs, each with a distinct composition. Indeed, all samples collected on the Nakivubo channel and some samples collected directly in the surrounding wetlands presented CST 1. This CST represents the prototypical composition of an area contaminated with wastewater with a diverse composition dominated by *Proteobacteria*, *Firmicutes* and *Bacteroidetes*. CST 2 was identified exclusively in samples collected in the wetlands, and the cluster-specific microbiome is highly dominated by environmental *Proteobacteria* making it representative of the natural microbial composition of the wetlands. CST 3 was identified in all samples collected in the inner Murchison Bay of Lake Victoria and the bacterial composition was largely dominated by *Microcystis aeruginosa*, a freshwater cyanobacterium, the presence of which indicates that potentially hazardous microbial contamination originating from wastewater is not permanently established in the lake. The heterogeneous microbial composition of wetland samples suggests that contamination from wastewater from the Nakivubo channel occurs throughout the wetlands, which might have important health-related implications for the local population. Hence, longitudinal monitoring of contamination levels throughout the system is warranted. In order to achieve a high resolution, the number of sampling points should be increased. However, the costs involved in running shotgun metagenomics on a large number of samples are prohibitive, and hence, cheaper alternatives are required. *E*. *coli* plating on agar cultures and subsequent counting is frequently used as the standard method to assess faecal-related contamination of water, and it has been shown previously that the counts of total faecal coliforms and *E*. *coli* in the Nakivubo wastewater system are above the WHO recommended thresholds that are 2.9 × 10^5^ and 9.9 × 10^4^ CFR per 100 ml, respectively^8^. However, these indicators are only quantitative, while information about the total bacterial diversity is also important. Our results indicate that, in addition to an above-threshold quantification of *E*. *coli* colonies, the intra-species diversity of *E*. *coli* (i.e. number of strains) is strongly correlated to the total number of bacterial strains in the Nakivubo ecosystem. This observation can be used to accurately estimate the total microbial diversity, and hence, potentially track wastewater contamination. The establishment of total bacterial diversity thresholds based on *E*. *coli* strain diversity, which would be indicative of wastewater contamination levels, will be helpful to provide a better description of the microbial diversity of the Nakivubo system. Importantly, such knowledge will contribute to a more precise profiling of associated health risks for exposed population groups, such as urban farmers and communities at risk of flooding events living in close proximity of the channel.

The second observation pertains to the sub-optimal capacity of the local sewage treatment plant (Bugolobi) to remove microbial contamination from wastewater at the time of sampling. Sampling locations C1 and C2 are located upstream and downstream, respectively, of the Bugolobi sewage treatment facility. Yet, the number of observed strains and the SDI decreased only slightly between C1 and C2, thus indicating a modest effect of the decontamination process on the microbial composition. Furthermore, we noted the introduction of several bacterial genera related to the sludge treatment process, including but not limited to the *Aminobacterium* or *Aminomonas* genera^26,27^, as well as several methanogens. Some bacterial genera, including *Erysipelothrix* or *Parasutterella*, which have been isolated from faecal material^28,29^, are also introduced in the sludge treatment process, hinting towards previous and potentially permanent contamination of the infrastructure. The causative agents of shigellosis, salmonellosis and yersiniosis, among others, decreased in abundance, while several other taxa known to contain human pathogenic species increased between both locations. Additional sampling is needed to assess whether this observation was punctual or is representative of the treatment capacity of the sewage treatment plant.

To identify potential leakages of the Nakivubo channel, we tested whether the taxonomic correlation of a wetland sample was highest with the closest channel sample. We showed that distance explains more than 90% of the taxonomic correlation for channel samples, suggesting a progressive shift of the bacterial communities along the wastewater channel. Based on this observation, we further compared these two metrics between wetland samples, which presented CST 1 with the channel samples. For three of the sampling points, namely W6, W8 and W10, the strongest correlation was observed between the bacterial composition and their closest sampling points on the wastewater channel. W6 and W8 were spatially closest to C4, while W10 was closest to C5. These observations suggest that containment of wastewater is insufficient around locations C4 and C5 on the Nakivubo channel and that leakage into the wetlands occurs around these points. This approach, combined with additional sampling points to increase resolution, could facilitate establishing an exact map of the system’s weaknesses.

With regard to the exact compositional differences between the three CSTs, samples presenting CST 1 are characterized by an overabundance of bacterial strains from the *Firmicutes*, *Bacteroidetes*, *Actinobacteria* and *Euryarchaeota* phylum, which are phyla commonly found in human feces^30,31^. *E*. *coli* overabundance in samples with CST 1 also hints towards the same faecal origin. We documented a high abundance of bacteria from the *Proteobacteria* phylum in CST 2, but that this overabundance is mainly attributable to environmental bacteria^32^. Samples collected in the inner Murchison Bay are characterized by the dominance of *Microcystis aeruginosa*, which is commonly found in freshwater bodies with warm temperatures, as it is the case in Lake Victoria.

There is strong evidence that the presence of pathogenic species identified in this study is directly associated to wastewater contamination. Indeed, several pathogenic species were found exclusively in wastewater samples or samples displaying similar taxonomic profiles. However, some taxa, including *Escherichia* spp., *Vibrio cholerae*, and *Klebsiella oxytoca*, were found in a majority of samples, probably due to the ubiquitous aspect of these bacterial species^33–36^.

Our study has several limitations that are offered for discussion. First, the study was conducted at the end of a dry period and did not include seasonal sampling. Such sampling would be more representative of long-term persistence of microbial communities in this environment and could potentially highlight additional features of the Nakivubo system. Taxonomic profiles were generated using a marker-based approach, which comprises specific limitations. For instance, the results presented in our study are specific to the profiling strategy described here and can vary when using a different database/profiler. Additionally – and as commonly observed in microbiome studies – there is a probability of false-positive annotation of reads. We sought to reduce this bias by limiting the results presented in this study to those with very high statistical confidence (e.g. *Q*-value or adjusted *P*-value < 0.01).

In conclusion, we present a proof-of-concept that system-wide microbiological characterization is possible using deep sequencing to gain a deeper understanding of a complex system, such as the Nakivubo wastewater system. While the resolution of the study is limited by the number of sampling locations and by the lack of temporally distinct sampling, the sequencing depth of each sample readily enabled us to highlight several novel key microbiological aspects of the Nakivubo channel, surrounding wetlands and the inner Murchison Bay. Four lessons can be drawn. First, based on the microbial composition, the system presented three distinct CSTs. Second, the Nakivubo channel has a clear impact on the wetland microbiota in specific locations. Third, the leakage of wastewater occurs mainly around two sampling locations. Fourth, several potentially harmful microorganisms for human health are found to be spread by wastewater contamination.

## Material and Methods

### Sampling strategy

**Figure 5:**
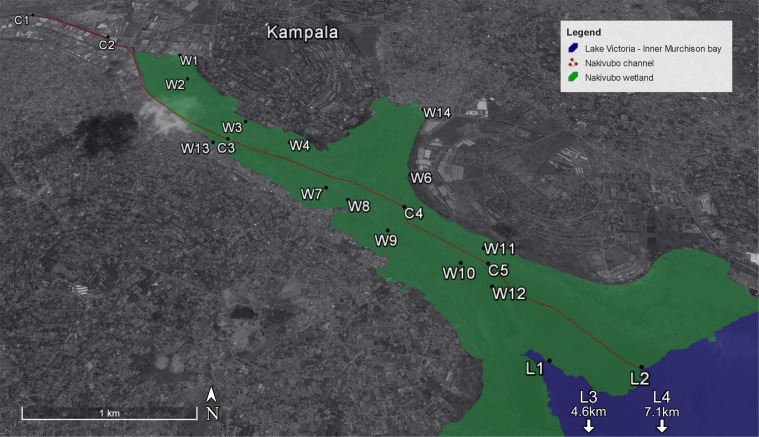
Map of the study area in Kampala, including the Nakivubo wetland area and the inner Murchison Bay (Lake Victoria). Samples collected on the channel are coded with C1–C5, samples collected in the wetlands are coded with W1–W14 and samples collected on Lake Victoria are coded with L1–L4. Map data: Google Earth Pro V7.1.8.3036 (June 20, 2017) with maps from DigitalGlobe (2017). Kampala, Uganda. 0°17′55.20″N, 32°37′51.78″E, Eye alt 4.73 km http://www.earth.google.com.

Water samples were collected at different locations distributed along the wastewater collection network of Kampala city, as shown in Fig. 5. Sample collection was performed after a prolonged dry period in October 2013 (Supplementary Table S3).

Samples were labelled as belonging to one of the three following ecosystems: (i) the Nakivubo wastewater channel (samples C1 to C5); (ii) the Nakivubo wetlands (samples W1–W14); and (iii) the inner Murchison Bay of Lake Victoria (samples L1–L4). Two samples from the W series (samples W13 and W14) were collected directly at informal communities’ outlets discharging into the wetlands. One sample from the L series (L3) was collected directly at the city’s freshwater intake, located in Gaba, approximately 5 km south from the Nakivubo channel outlet. Sample L4, located in the middle of the Murchison Bay, approximately 7.5 km south from the Nakivubo outlet, serves as a low human-related contamination control. Geographical coordinates were obtained with a hand-held global positioning system (GPS; Garmin, Olathe, KS, USA). Sampling locations are available in Supplementary Table S4 and detailed information on general microbial and chemical water quality have been reported elsewhere^8^.

### Sample collection procedure, storage and nucleic acid extraction

At each location, 2–3 l of water were collected at a depth of 0–20 cm in sterile containers. Samples were kept in a cooling box until arrival in the laboratory, stored in a fridge at a temperature of 4 °C and immediately concentrated. Concentration was performed using a benchtop tangential flow filtration unit (Natan GmbH; Zurich, Switzerland) and UP020 20 kDa filter membranes (Microdyn-Nadir; Wiesbaden, Germany) into 50 ml sterile tubes. The concentrated samples were frozen at −20 °C and transferred to Switzerland in a cooling box to prevent thawing and avoid microbial growth. Upon arrival in Switzerland, the samples were further concentrated with Amicon Ultra Centrifugal Filter Units with a molecular cut-off of 10 kDa (Millipore; Billerica, MA, USA) into a smaller volume of approximately 250–300 µl. One sample (sample W5) was lost during processing and was omitted from the analysis.

Nucleic acids were isolated from 250 µl of the concentrated samples using a PowerSoil DNA isolation kit (MO-BIO; Carlsbad, CA, USA) following the manufacturer’s instruction except for the elution step which was done in 60 µl purified water. Extracted samples were quantified on a Qubit 2.0 fluorimeter using the dsDNA high-sensitivity assay (Life Technologies; Darmstadt, Germany).

### Sequencing and data analysis

DNA libraries were prepared from 30 µl of the different samples using NEBNext Ultra library preparation kits (New England Biolabs; Ipswich, MA, USA). Samples were pooled on an Illumina HiSeq 2500 (Illumina; San Diego, CA, USA) in 2 × 125 base pairs (bp) paired-end mode for sequencing. An in-house developed Perl pipeline was used to automatize the dataset analysis in two steps, namely: (i) a pre-processing step of the raw sequences datasets; and (ii) the comparison of curated reads to a genome-specific database, as described previously^37^.

Pre-processing of the raw datasets was further divided into two sub-steps, including: (i) a quality control; and (ii) a quality filtering of raw sequences. The tool FastQC version 10.1^38^ was used to assess the overall sequencing quality and the software suite ea-utils version 1.1.2^39^ was employed to remove the reads not passing the CASAVA filter from Illumina. The same tools were used to remove bases with a quality score below Q20 at both 5′ and 3′ ends.

The second step of the analysis involved a comparison of sequenced reads to a database containing strain-specific markers for 4,088 bacterial strains (GSMer^40^). The comparison was performed using the BLAST software version 2.2.28+^41^. We used the BLASTn algorithm with a wordsize of 50 which equals the length of the markers in the GSMer database, and hence, only allow perfect hits (100% similarity with no gaps allowed), to ensure accurate taxonomic annotation of the reads. A minimum cut-off of two hits in two markers per strain was used to include the strain in the taxonomic profiles. The complete taxonomic information for each BLASTn hit was retrieved using the NCBI taxon identifier (taxid) and the corresponding BioPerl version 1.2.9 modules^42^.

### Statistical analysis

Hierarchical clustering was performed using the OriginPro 2017 suite (OriginLab Corporation; Northampton, MA, USA). The list of edges for the network analysis was generated using the R platform (R Development Core Team, 2011) with the package “igraph”^43^ and vizualised using Gephi version 0.9.1^44^. Distances were calculated between each location using the R package “geosphere”^45^. Alpha diversity indices (Shannon diversity and strain richness) were calculated at the strain level using the software PAST3^46^. Linear regression analyses were performed with XLSTAT 2019 (Addinsoft; Paris, France). Taxonomic profiles were compared with Lefse version 1.0^47^, which performs a Kruskal-Wallis test and uses a linear discriminant analysis to rank statistically significant differences. Group medians comparisons were performed using Kruskal-Wallis tests. *P*-values were corrected (=*Q*-values) for multiple testing bias using the Benjamini-Hochberg adjustment^48^ in STAMP version 2.1.5^49^, as appropriate. Throughout the study, statistical significance is reached with a *Q*-value threshold below 0.01.

## Supplementary information

Supplementary information

Supplementary Table 2

## Data Availability

Sequence data that support the findings of this study have been deposited in the NCBI Short Read Archive with the primary accession code SRP127721.
