# Supplementary material for: Qualitative microbiome profiling along a wastewater system in Kampala, Uganda
Source: Sci Rep. 2019 Nov 22;9:17334. doi: 10.1038/s41598-019-53569-5 (PMC6874685; doi:10.1038/s41598-019-53569-5)
Supplement: Supplementary file 1 — Supplementary information [file 41598_2019_53569_MOESM1_ESM.pdf]

Supplemental information belonging to:

Qualitative microbiome profiling along a wastewater system in Kampala, Uganda

Pierre H.H. Schneeberger, Samuel Fuhrimann, Sören L. Becker, Joël F. Pothier, Brion Duffy, Christian Beuret, Jürg E. Frey and Jürg Utzinger

Supplementary Tables

- **Supplementary Table 1**. Sample metadata and bioinformatics metrics.
- **Supplementary Table 2**. Abundance table of bacterial taxa (GSMer).
- **Supplementary Table 3.** Daily rainfall during the month preceding sample collection.
- **Supplementary Table 4.** Geographical coordinates and abbreviations of collected samples.

Supplementary Figures

- **Supplementary Figure 1**. Relations between distance (D) and bacterial composition (BC).

Supplementary Table 1. Sample metadata and bioinformatics metrics. This table details the amount of DNA obtained for each sample and the most important metrics generated during the *in silico* analyses. [] = concentration; ng = nanogram; µl = microlitre.

**Sample**	**[] (ng/µl)**	**total (ng)**	**nr of queries**	**nr hits genome-specific database (GSMer)**
Channel 1	6.92	415.2	76108948	36139
Channel 2	3.47	208.2	66389226	20326
Channel 3	1.81	108.6	60020276	18807
Channel 4	2.02	121.2	48833748	11074
Channel 5	4.48	268.8	44912594	9333
Wetland 1	1.78	106.8	47309246	2225
Wetland 2	7.56	453.6	54497660	831
Wetland 3	1.77	106.2	83569972	30127
Wetland 4	6.12	367.2	58422032	365
Wetland 6	4.84	290.4	42986736	570
Wetland 7	11.3	678	58987970	4098
Wetland 8	2.44	146.4	43258762	983
Wetland 9	2.22	133.2	44057282	820
Wetland 10	9	540	37016806	4290
Wetland 11	19.2	1152	52543330	277
Wetland 12	18.2	1092	47822670	128
Wetland 13	4.52	271.2	43188180	16121
Wetland 14	7.72	463.2	52635258	1617
Lake 1	3.23	193.8	139398288	40016
Lake 2	5.8	348	44112472	40130
Lake 3	3.35	201	48621746	12852
Lake 4	1.86	111.6	45562626	3796

Supplementary Table 3. Daily rainfall during the month preceding sample collection. Days of sampling are highlighted in bold.

Day	Daily rainfall (mm)	Day	Daily rainfall (mm)
15/08/2013	0.5	14/09/2013	0.0
16/08/2013	0.0	15/09/2013	20.5
17/08/2013	3.3	16/09/2013	0.0
18/08/2013	0.0	17/09/2013	N.A
19/08/2013	0.0	18/09/2013	3.0
20/08/2013	1.2	19/09/2013	9.5
21/08/2013	0.6	20/09/2013	0.7
22/08/2013	0.0	21/09/2013	22.5
23/08/2013	0.1	22/09/2013	7.0
24/08/2013	0.0	23/09/2013	0.2
25/08/2013	2.5	24/09/2013	0.5
26/08/2013	0.0	25/09/2013	0.0

27/08/2013	0.0	26/09/2013	N.A
28/08/2013	0.0	27/09/2013	0.0
29/08/2013	0.0	28/09/2013	8.6
30/08/2013	5.4	29/09/2013	27.5
31/08/2013	27.6	30/09/2013	15.3
01/09/2013	27.6	01/10/2013	0.0
02/09/2013	51.9	02/10/2013	10.6
03/09/2013	4.0	03/10/2013	2.0
04/09/2013	0.0	04/10/2013	0.0
05/09/2013	0.0	05/10/2013	0.0
06/09/2013	0.0	06/10/2013	N.A
07/09/2013	0.3	07/10/2013	1.8
08/09/2013	37.0	08/10/2013	0.2
09/09/2013	0.1	09/10/2013	0.0
10/09/2013	0.6	10/10/2013	0.6
11/09/2013	0.1	**11/10/2013**	0.3
12/09/2013	0.0	**12/10/2013**	0.0
13/09/2013	0.0	**13/10/2013**	0.0

Supplementary Table 4. Geographical coordinates and abbreviations of collected samples. This table shows coordinates of sampling locations used in this study and the corresponding abbreviations used in the manuscript.

Sample name	Abbreviation	Latitude	Longitude
Channel 1	C1	0.318048	32.602841
Channel 2	C2	0.315294	32.60931
Channel 3	C3	0.304312	32.619833
Channel 4	C4	0.298289	32.631575
Channel 5	C5	0.293887	32.63647
Lake 1	L1	0.287377	32.639302
Lake 2	L2	0.286987	32.644142
Lake 3	L3	0.241954	32.642259
Lake 4	L4	0.219695	32.638197
Wetland 1	W1	0.313125	32.615092
Wetland 10	W10	0.293929	32.634873
Wetland 11	W11	0.295019	32.636289
Wetland 12	W12	0.292232	32.636585
Wetland 13	W13	0.303999	32.61899
Wetland 14	W14	0.307199	32.632897
Wetland 2	W2	0.310438	32.616108
Wetland 3	W3	0.305992	32.620805
Wetland 4	W4	0.303986	32.623959
Wetland 6	W6	0.301028	32.631979
Wetland 7	W7	0.29989	32.626637
Wetland 8	W8	0.298867	32.628057

Wetland 9	W9	0.296385	32.630572

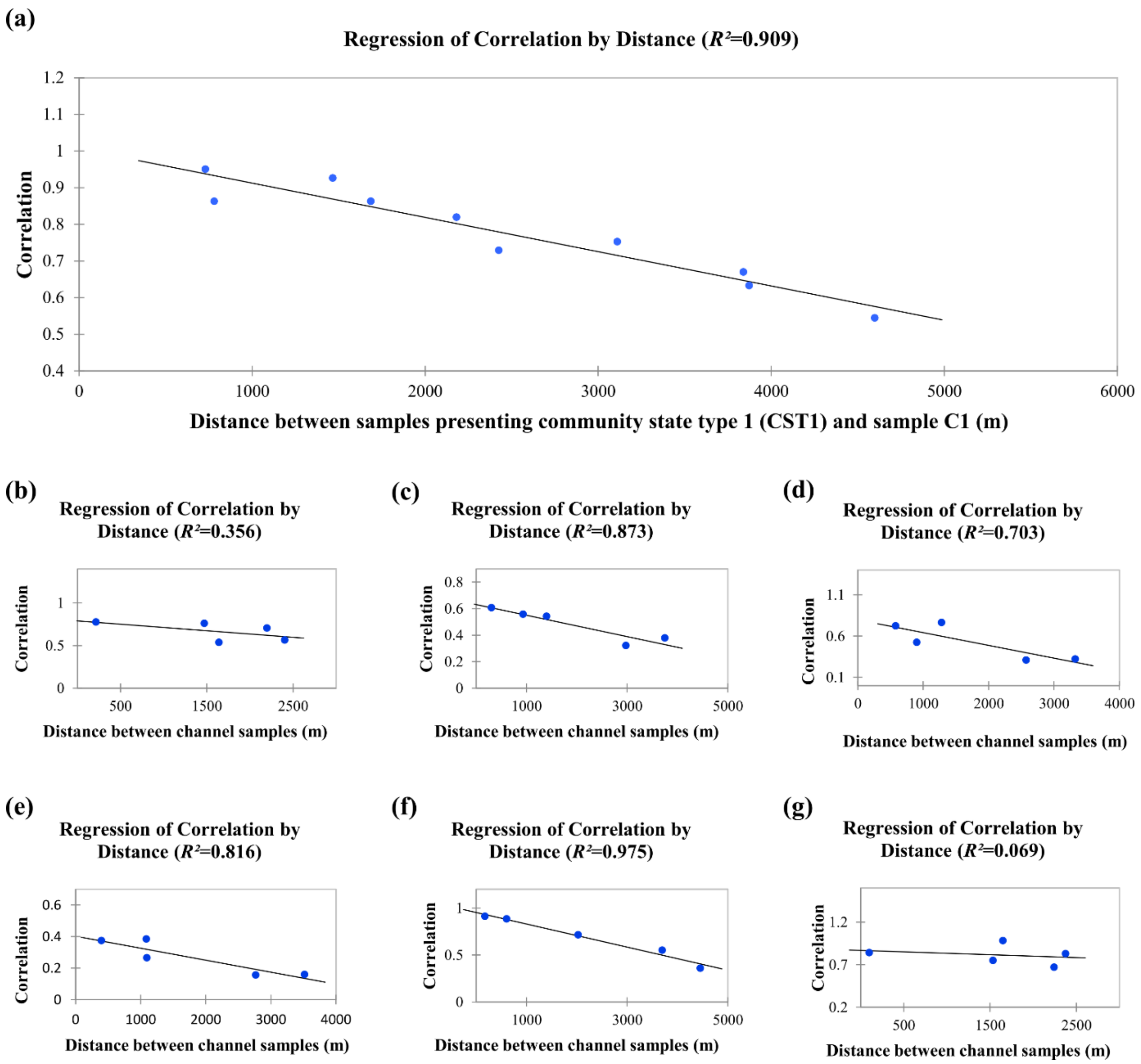

Supplementary Figure 1. Relations between distance (D) and bacterial composition (BC). (**a**) Linear regression analysis of taxonomic correlation and distance between each channel samples. (R^2 = 0.909, $P < 10^{-4}$). The taxonomic correlation between samples can be estimated, along the wastewater channel, using the following equation: BC = 1.006 – $9.368\times10^{-5}\times$D. (**b-g**) represent the taxonomic correlation between wetland samples presenting community state type 1 (CST 1) (W3, W6, W7, W8, W10 and W13, respectively) and their distance to the channel samples. A high

R^2 indicates that the taxonomic profile is the most closely related to its closest channel sampling location. P = 0.29, 0.02, 0.076, 0.035, 0.002 and 0.669, respectively.
